# Effect of Probiotic *Limosilactobacillus reuteri* and Hyaluronic Acid Gel Combination Therapy on Neutrophil Response, Neovascularization, and Reepithelialization in the Wound-Healing Process Following Tooth Extraction: An In Vivo Study

**DOI:** 10.1055/s-0045-1813034

**Published:** 2026-03-03

**Authors:** Armelia Sari Widyarman, Desak Made Hari Wijayanti, Melanie Hendriaty Sadono, Firstine Kelsi Hartanto, Komariah, Wiwiek Poedjiastoeti

**Affiliations:** 1 Department of Oral Biology, Faculty of Dentistry, Universitas Trisakti, Kota Jakarta Barat, Indonesia; 2 Faculty of Dentistry, Universitas Trisakti, Kota Jakarta Barat, Indonesia; 3 Department of Oral Medicine and Oral Pathology, Faculty of Dentistry, Universitas Trisakti, Kota Jakarta Barat, Indonesia; 4 Department of Oral and Maxillofacial Surgery, Faculty of Dentistry, Universitas Trisakti, Kota Jakarta Barat, Indonesia

**Keywords:** *Limosilactobacillus reuteri*, hyaluronic acid, probiotic, neutrophils, neovascularization, reepithelialization, wound healing

## Abstract

**Objective:**

This study aimed to analyze the effects of supportive therapy using a combination of *Limosilactobacillus reuteri (L. reuteri)* and hyaluronic acid (HA) gel on the wound-healing process during inflammatory, proliferative, and remodeling/maturation phases following tooth extraction.

**Materials and Methods:**

Forty-five white Wistar rats ( *Rattus norvegicus* ) were divided into five treatment groups after the left mandibular central incisor was extracted. The experimental groups were treated with HA gel, probiotic *L. reuteri* gel, a combination of HA gel and *L. reuteri* gel, and povidone-iodine (PVP-I). The negative control group was treated with sterile tampons. Tissue samples were collected on days 3, 7, and 14 post-tooth extractions and analyzed histologically using hematoxylin–eosin (HE) staining to evaluate neutrophil numbers, neovascularization, and epithelial thickness.

**Statistical Analysis:**

Data analysis was performed using IBM SPSS version 29. Inter-rater reliability was tested using the intraclass correlation coefficient (ICC), followed by normality and homogeneity tests. For normally distributed data, parametric hypothesis testing was conducted using one-way ANOVA at a 95% confidence level ( *p* < 0.05). The post hoc least significant difference test was used to observe significant differences between the groups.

**Results:**

Significant differences in neutrophil numbers, neovascularization, and re-epithelialization were found between the negative control and combination group ( *p* < 0.05) on days 3, 7, and 14.

**Conclusion:**

HA and *L. reuteri* combination therapy is effective in accelerating wound healing after tooth extraction.

## Introduction

Oral and dental diseases are prevalent worldwide, including in Indonesia. The 2018 Basic Health Research (RISKESDAS) report indicated a prevalence of 57.6% for oral health problems. 1 Tooth extraction is still one of the most common dental procedures; national data indicate that approximately 7.9% of the population has undergone tooth extraction, with dental caries being the leading cause (42.8%). 2 After tooth extraction, wound healing is initiated. However, the complexity of the oral cavity, as an ecosystem containing diverse microorganisms, poses a risk of post-extraction complications. Moreover, the prolonged healing period of the socket often causes significant patient complaints, as it interferes with mastication, phonetics, and aesthetics. Therefore, several studies have investigated supportive materials to accelerate wound healing following tooth extraction. 3

Immediately after a wound occurs, blood clotting takes place, followed by an acute inflammatory response. Neutrophils migrate to the damaged tissue and perform phagocytosis of microorganisms, foreign bodies, and necrotic tissue. 4 The neutrophils are then replaced by monocytes that move from the microcirculation to the wound site, where they differentiate into macrophages, initiating the final inflammatory process within 46 to 72 hours of injury. 4 5 Once inflammation subsides, the proliferative phase plays a crucial role in closing the wound (reepithelialization), restoring blood vessel tissue, and forming granulation tissue. 6 During this phase, proper wound healing is characterized by the appearance of a large number of fibroblasts, collagen, and new blood vessels. 3

Wound healing is influenced by several internal factors, including the patient's age, systemic health conditions such as diabetes mellitus, nutritional status, immune response, and habits such as smoking, all of which can significantly affect the rate and quality of healing following tooth extraction. 5 The external factors include the application of a 0.9% NaCl solution to the socket, the use of antiseptic povidone–iodine (PVP-I) at concentrations of 1 or 2%, or antibiotics to prevent infection. 7 8 9 Another therapeutic option is hyaluronic acid (HA) gel, which is known to promote wound healing following tooth extraction. HA has been shown to accelerate reepithelialization and stimulate changes in protein expression *in vivo* in human dermal incision wounds. However, its effect on the inflammatory process is not significant, as indicated by erythema assessment in a previous study. 10

Probiotics are widely used as supportive therapy and have been shown to reduce inflammation in the oral mucosa. One probiotic bacterium frequently utilized for oral health is *Limosilactobacillus reuteri (L. reuteri)* , which produces reuterin, an antimicrobial agent that supports wound healing. 11 12 However, studies evaluating the role of probiotics in wound healing *in vivo* are limited, and further research is necessary. 13 Previous *in vitro* studies have demonstrated that *L. reuteri* can promote the migration, proliferation, and osteogenic differentiation of gingival mesenchymal stem cells, thereby accelerating the wound-healing process. 14 These findings provide a strong biological rationale for investigating the effects of *L. reuteri in vivo* , particularly when combined with other regenerative agents such as HA, which may synergistically enhance tissue repair and wound healing. To our knowledge, no studies have examined the combination of HA and the probiotic *L. reuteri* , administered at closely timed intervals, on wound healing following tooth extraction. This study evaluates several wound-healing phases by assessing neutrophil count, blood vessel formation, and epithelial thickness. We hypothesize that the combination of HA and *L. reuteri* is effective in accelerating tissue repair after tooth extraction, thereby helping to alleviate patient complaints.

## Materials and Methods

This was an in vivo animal experimental study with a randomized post-test only control group design.

### Sample

Forty-five healthy (as indicated by active movement) male Wistar rats ( *Rattus norvegicus* ), aged 3 to 4 months and weighing 200 to 260 g, were used in this study. The minimum sample size per group was calculated using the formula for analytical studies with numerical data:



where:

- *n* 1 = number of samples in the treatment group,
- *n* 2 = number of samples in the comparison group,
- *Zα* = 1.96 (standard normal deviate for *α* = 5%),
- *Zβ* = 1.96 (standard normal deviate for *β* = 20%),
- *
X _1_ –X _2_* = 156.0–129.8 (minimal difference consideration meaningful),
- *S* = 5.25 (pooled standard deviation, determined using the pooled standard deviation formula from the previous study). 15

The result calculation resulted in *n* = 0.58 per group, which has been rounded up to 1 rat as the minimum requirement. To minimize the risk of sample loss and ensure reliable observation data, three rats were included per group at each observation time point. Observations were conducted on days 3, 7, and 14, resulting in a total of 45 rats.

Rats were randomly assigned to groups using a simple randomization method, ensuring three animals per group at each time point.

This study was conducted in accordance with 3R principles (replacement, reduction, and refinement) to minimize animal use. 16

### Probiotic Gel Preparation

Probiotic gel was prepared from Interlac Pro-D tablets (800 mg; BioGaia AB, Stockholm, Sweden) in a pharmaceutical laboratory. The tablets, containing *L. reuteri* strains DSM 17938 and ATCC PTA 5289, were dissolved and formulated into a gel suitable for topical application in the extraction socket. Carboxymethyl cellulose sodium (CMC–Na) was used as the base material. 17 A total of 1.5 g of CMC–Na powder was dissolved in 50 mL of distilled water, heated to 70 °C, and left to sit for 15 minutes. Then, 50 mL of cold water was added, and the mixture was stirred with a magnetic stirrer until it expanded. Next, 1.75 g of CMC–Na was placed in a beaker, and 3.15 mL of glycerin, 0.3 mL of propylene glycol, 45 mL of distilled water, and 800 mg (two tablets) of Interlac Pro-D were added. The solution was then stirred until it became homogeneous. To ensure that the bacteria in the gel remained viable, an *L. reuteri* culture was performed in the microbiology laboratory using de Man–Rogosa–Sharpe (MRS) agar under anaerobic conditions and incubated for 48 hours. 17 18

### Tooth Extraction and Therapy Administration

The treatment of the animals was supervised by veterinarians at the Animal Hospital of Udayana University, Bali. The rats underwent a 7-day adaptation period in wire mesh cages with bedding made of wood shavings. They were provided with food and water ad libitum and were continuously observed until the completion of the study.

The left mandibular central incisor was extracted using a scalpel and a needle holder under general anesthesia with ketamine (50–80 mg/kg; Kepro B.V., Deventer, the Netherlands) and xylazine (20 mg/kg; Agrovet Market, Lima, Peru). After tooth extraction, the depth of the socket was measured with a periodontal probe to ensure that the treatment material was applied to the entire socket area and to determine the appropriate volume of gel. Treatment was administered once daily, with a volume of 0.1 mL of HA gel, probiotic gel, and PVP-I. In the T3 group, HA gel was applied immediately after tooth extraction, followed by *L. reuteri* probiotic gel approximately 30 minutes later. This delay was intentionally implemented to allow sufficient time for the HA gel to adhere to and be partially absorbed by the socket tissue, creating a conducive, moist, and biocompatible environment for the wound-healing process. The delayed probiotic application aims to enhance colonization potential, as HA has been reported to inhibit the adhesion of pathogenic bacteria without negatively affecting the viability or activity of beneficial strains such as *L. reuteri* . 14 19

### Histopathological Examination

For tissue preparation, the left mandible was cut under general anesthesia as specified above on days 3, 7, and 14 after tooth extraction, and euthanasia was performed using a lethal dose of ketamine. The tissue samples were fixed in 10% neutral-buffered formalin to prevent necrosis and sent to a laboratory for hematoxylin–eosin (HE) staining to enable the observation of neutrophils, blood vessels, and epithelial tissue. The samples were examined using a binocular light microscope at 400× magnification with three different fields of view. 20 21 The examination was performed by three interpreters, beginning with cell calibration and observation using Fiji ImageJ software. The clinical characteristics of neutrophils, blood vessels, and epithelial cells were observed, and cell counts were performed in each field of view. The total cell count from all fields of view was recorded and expressed as cells per high-powered field (HPF) or cells per three fields of view (cells/3HPFs) for each cell type. 22 Subsequently, the average number of cells counted by the three interpreters was calculated to ensure consistency and accuracy. This methodology ensured that the histopathological analysis provided reliable data on wound healing in the different groups. 21 22

### Data Analysis

Data analysis was performed using IBM SPSS Statistics version 29 (IBM Corp., Armonk, New York, United States). The cell counts were obtained from observations by three interpreters. Inter-rater reliability was assessed using intraclass correlation coefficients (ICCs) to evaluate consistency and reliability between interpreters. For normally distributed data, parametric hypothesis testing was performed using one-way ANOVA at a 95% confidence level ( *p* <0 0.05). In cases of significant differences, the post hoc least significant difference (LSD) test was used to determine which groups exhibited significant differences. For abnormally distributed data, nonparametric hypothesis testing was performed using the Kruskal–Wallis test, followed by the post hoc Mann–Whitney test to assess pairwise differences. In both tests, values of *p* < 0.05 were considered statistically significant.

## Results

**Fig. 1:**
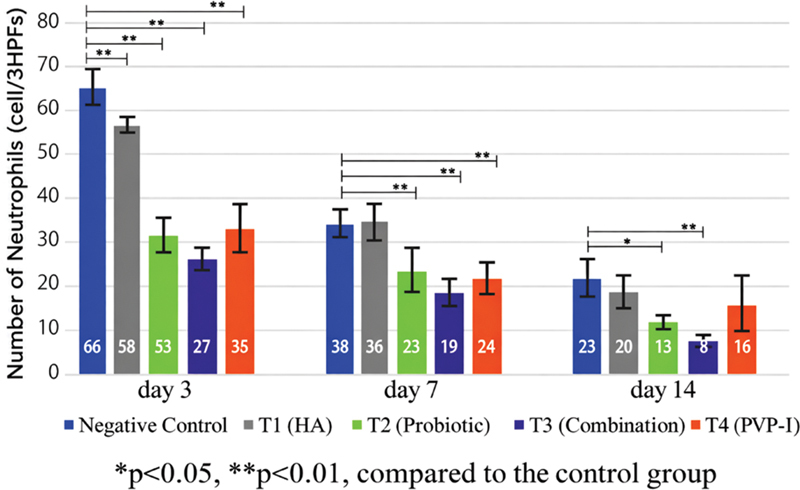
Graph of the average neutrophil count in each group on days 3, 7, and 14 in histopathological examination. NC (negative control), T1 (HA), T2 (probiotic), T3 (combination of HA and probiotic), T4 (PVP-I); * *p* < 0.05, ** *p* < 0.01 compared to the NC.

**Fig. 2:**
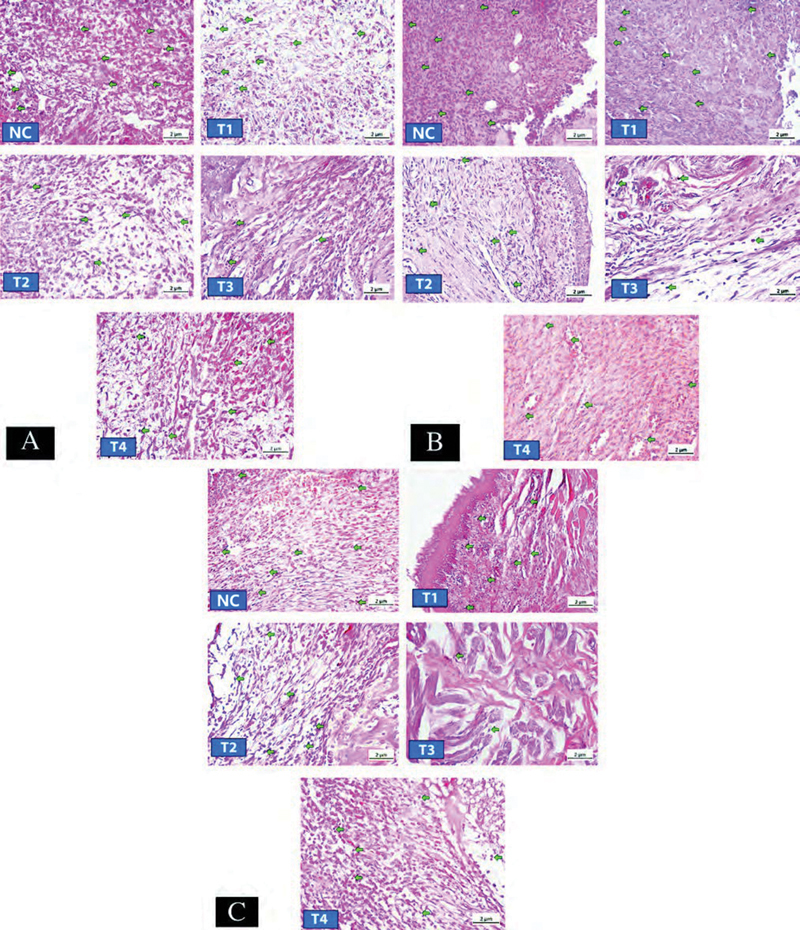
Histopathological features of the number of neutrophils marked by green arrows. ( **A** ) Day 3, ( **B** ) day 7, ( **C** ) day 14. NC (negative control), T1 (HA), T2 (probiotic), T3 (combination of HA and probiotic), T4 (PVP-I).

**Fig. 3:**
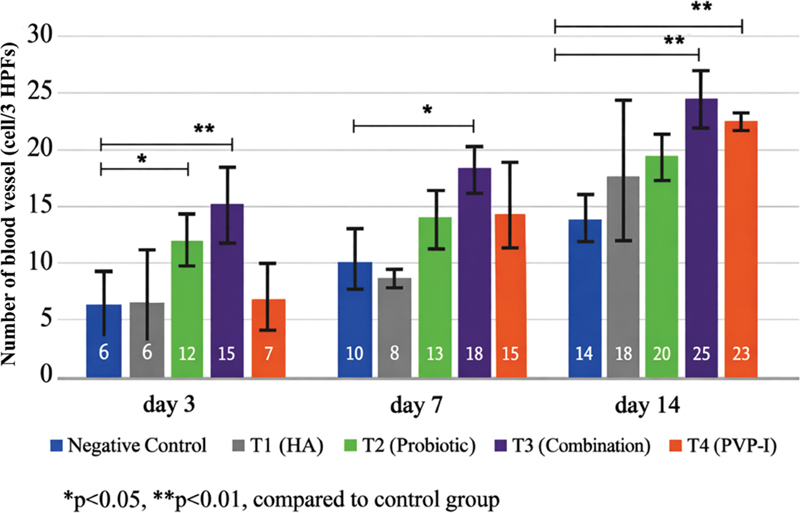
Graph of the average number of blood vessels in each group on days 3, 7, and 14 in histopathological examination. NC (negative control), T1 (HA), T2 (probiotic), T3 (combination of HA and probiotic), T4 (PVP-I), * *p* < 0.05, ** *p* < 0.01 compared to the NC.

**Fig. 4:**
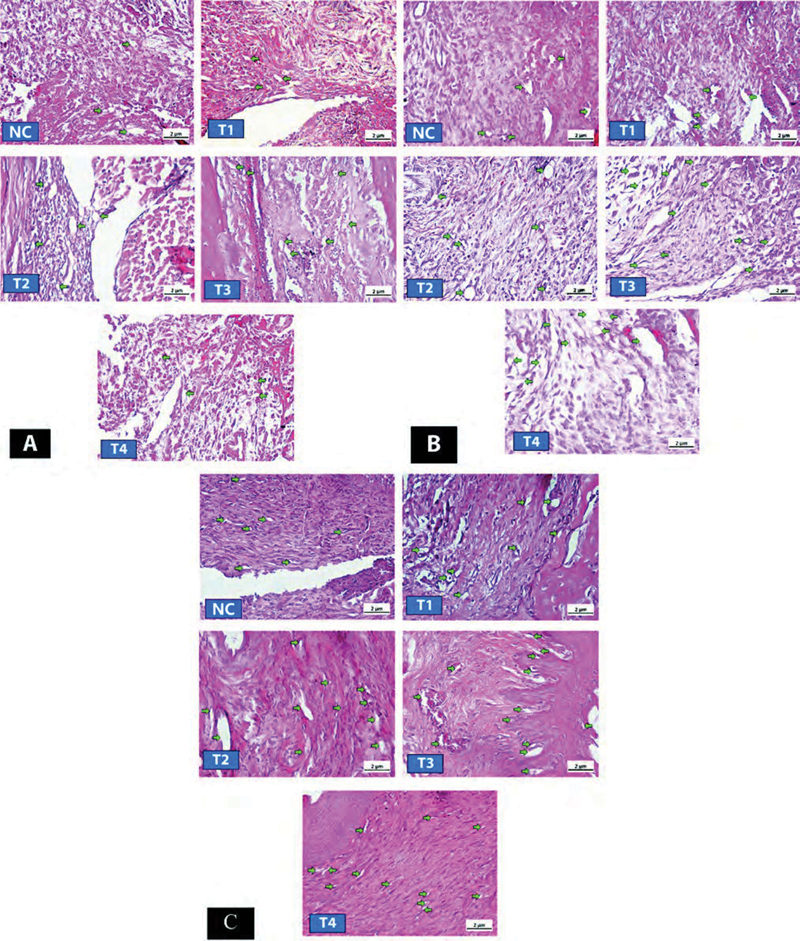
Histopathological features of the number of new blood vessels marked by green arrows. ( **A** ) Day 3, ( **B** ) day 7, ( **C** ) day 14. NC (negative control), T1 (HA), T2 (probiotic), T3 (combination of HA and probiotic), T4 (PVP-I).

**Fig. 5:**
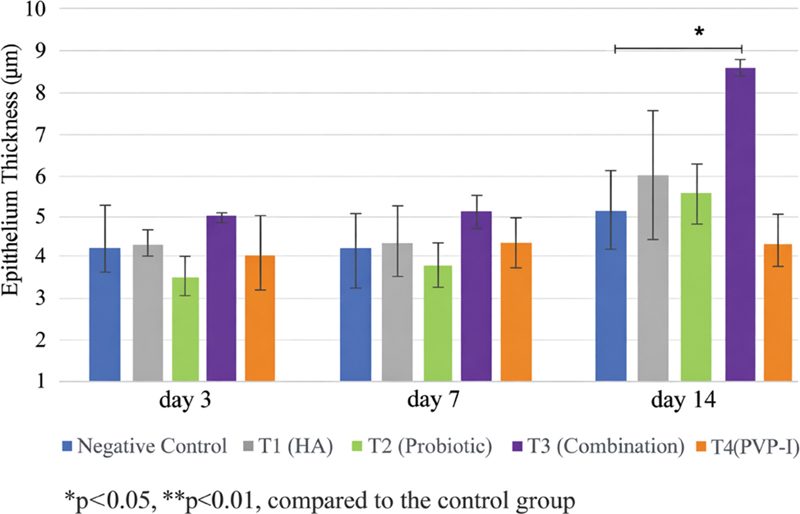
Graph of the average epithelial thickness count in each group on days 3, 7, and 14 in histopathological examination. NC (negative control), T1 (HA), T2 (probiotic), T3 (combination of HA and probiotic), T4 (PVP-I), * *p* < 0.05, ** *p* < 0.01 compared to the NC.

**Fig. 6:**
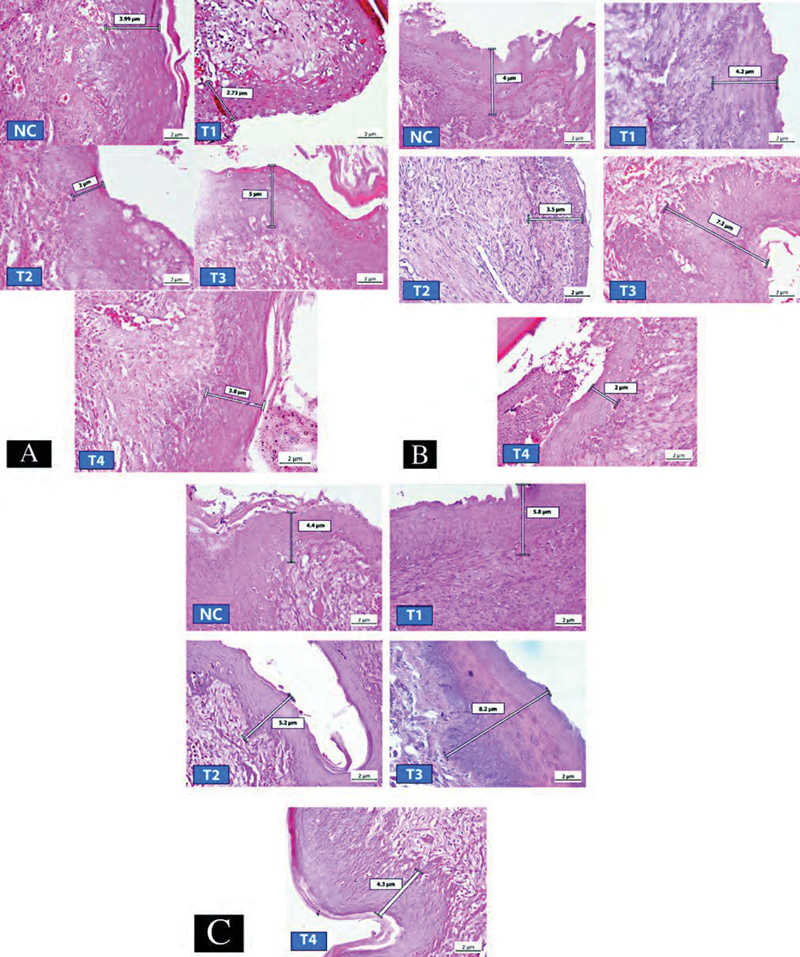
Histopathological features of the average epithelial thickness. ( **A** ) Day 3, ( **B** ) day 7, ( **C** ) day 14. NC (negative control), T1 (HA), T2 (probiotic), T3 (combination of HA and probiotic), T4 (PVP-I).

Histopathological analysis results of Wistar rats' tooth socket following treatment with a combination of HA and *L. reuteri* revealed the tissue features and cell counts shown in Figs. 1 to 6 , using HE staining.

All animals completed the treatment protocol without mortality or exclusion from the analysis. Histological samples were successfully collected and analyzed from 45 rats' socket tissues at each observation time point (days 3, 7, and 14).

## Discussion

After tooth extraction, the primary concern is the speed of wound healing. This process continues until the socket is fully closed, and tissue repair continues for approximately one year. If not properly managed, tooth extraction can cause complications that may result in prolonged inflammation with an unpredictable healing timeline. Wound closure and tissue healing are also influenced by the size and depth of the wound. 17 23

The therapeutic material used in this study was in the form of gel, which is easy to apply to the sockets of animal subjects and potentially human patients. The use of a topical gel formulation in this study holds clinical relevance, especially for wound healing in the oral cavity. Local application allows the therapeutic agent, *L. reuteri* and HA, to be delivered directly to the wound site, achieving higher local concentrations while minimizing systemic absorption or side effects. The gel-based delivery system demonstrated several clinical advantages. It adheres more effectively to the oral mucosa surface, allowing prolonged contact time between the bioactive compounds and the wound tissue. 24

A daily application volume of 0.1 mL of gel was selected based on the average depth of the extraction sockets. This dosage was considered appropriate for achieving full socket coverage while minimizing overflow. The comparability and relevance of this volume were supported by a similar dosage (0.16 mL/day) reported in a previous study. 24 25

In this study, the significantly lower ( *p* < 0.05) average neutrophil count in the T3 (combination therapy) group than in the other groups on day 3 indicated that acute inflammation had begun to subside. This was followed by a decrease in neutrophil counts in the T1 (probiotic) and T4 (PVP-I) groups. Neutrophils are typically present at the trauma site during the first 6 to 24 hours, peaking on day 3, and play an important role in phagocytosing necrotic tissue and preventing infection. 26 On day 7, all groups showed a decrease in the number of neutrophils, which is in line with the general theory of wound healing. This decrease typically occurs when the wound heals without complications. 21 However, in this study, there was a statistically significant difference ( *p* < 0.05) on day 7 between the T3 group and the NC (negative control) group, indicating a significant reduction in inflammation in the former group. Histopathological observations ( Fig. 1 ) revealed that inflammatory cells were still present in the control, T1, T2, and T3 groups. However, on day 14, the neutrophil count in the T3 group was considerably lower than in the other groups, suggesting that the HA and *L. reuteri* combination therapy led to a more rapid reduction in inflammation. A rapid subsidence of inflammation indicates the absence of excess pathogenic bacteria that could interfere with wound healing. 27 In a previous study, the application of HA gel did not significantly reduce inflammation. 10 In this study, the combination of HA and probiotic *L. reuteri* resulted in a greater reduction in the number of inflammatory neutrophil cells on days 3, 7, and 14 than HA alone.

The next process typically occurring in response to injury or tissue growth is neovascularization. Neovascularization is based on two processes: angiogenesis, which is the formation of new blood vessels from preexisting vessels, and vasculogenesis, which is the creation of new blood vessels from endothelial progenitor cells. In this study, neovascularization was analyzed on days 3, 7, and 14 after tooth extraction by observing the number of blood vessels in the socket area. The proliferative phase involves epithelialization, angiogenesis, granulation tissue formation, and collagen deposition and occurs from day 4 to day 14 after injury. 26 In this study, neovascularization was observed in all treatment groups, as shown in the graph ( Fig. 3 ). The T3 group exhibited a higher average number of blood vessels on days 3, 7, and 14 than the other groups. The number of blood vessels on all observation days was significantly higher in the T3 group than in the T1 and control groups. This suggests that the administration of *L. reuteri* alone had a positive effect similar to that of the combination therapy on neovascularization.

Reepithelialization was also documented on days 3, 7, and 14 after tooth extraction by measuring the thickness of the epithelium formed in the socket area. In line with previous evidence suggesting that probiotics can promote the proliferation and migration of gingival epithelial cells both in vivo and in vitro, the *L. reuteri* –HA combination therapy promoted re-epithelization. Probiotics can also modulate the level of inflammation at the wound site and accelerate epithelial healing due to their ability to downregulate the proinflammatory cytokines IL-1β and TNF-α in damaged gingival epithelial tissue. 28 29 Furthermore, probiotics act against pathogenic microorganisms in the wound area and can regulate endocrine levels, thereby promoting wound healing. 29 In line with these findings, as shown in the graph of average epithelial thickness in Fig. 5 , the HA– *L. reuteri* combination therapy resulted in higher epithelial thickness on days 3, 7, and 14 than the other treatments. In Fig. 6C , epithelial tissue is clearly visible in the socket area in the T3 group on day 14. On days 3 and 7, epithelial thickness did not differ significantly between the T1 and T3 groups, indicating that the HA and combination treatments had similar effects on reepithelialization up to day 7. However, on day 14, the T3 group exhibited significantly greater ( *p* < 0.05) average epithelial thickness than the T1 group, indicating that the combination treatment promoted more rapid reepithelialization during the cell proliferation and maturation phases. In comparison, epithelial formation progressed more slowly in the NC group. Moreover, in the T4 group, which received PVP-I (primarily used as an antimicrobial in this study), no modulation of epithelial formation was observed.

The combination of *L. reuteri* and HA in this study demonstrated enhanced therapeutic outcomes compared to single-agent treatments, suggesting a potential synergistic effect. HA is known for its ability to modulate inflammation, support cellular migration, and retain moisture at wound sites, creating an optimal healing environment. *L. reuteri* provides anti-inflammatory, antimicrobial, and immunomodulatory effects by regulating cytokine expression and promoting epithelial regeneration. Their combined application may accelerate the resolution of inflammation, enhance neovascularization, and promote epithelial healing more effectively than either used alone. 30 31

This study has several limitations. The observation period was limited to 14 days, which may not capture the complete remodeling phase of wound healing. Additionally, only histopathological outcomes were assessed; molecular or microbiological parameters were not explored. Future research should involve clinical trials in human subjects to validate the efficacy of HA– *L. reuteri* therapy. It would also be beneficial to explore different dosages, delivery methods, and the molecular mechanisms underlying the observed effects. Investigating long-term outcomes and potential synergistic effects with other therapeutic agents could further refine its clinical applications. Moreover, the development of a single, integrated gel formulation that combines both HA and probiotic components may improve clinical practicality by simplifying application procedures and enhancing patient compliance. This concept represents an initial innovation that could serve as a foundation for future formulation research and development of supporting therapies for post–tooth extraction wound healing.

## Conclusion

In this study, histopathological observations of neutrophil activity, neovascularization, and reepithelization demonstrated that the combination therapy of HA and *L. reuteri* significantly accelerated the wound-healing process following tooth extraction. Compared to both the negative control and the commonly used PVP-I treatment, the HA and *L. reuteri* gel promoted a more favorable healing response, indicating its potential effective post–tooth extraction therapeutic option.

**Table 1:** Experimental groups and respective treatment protocols

Group	Treatment
NC (negative control)	Received no treatment (medicament)
T1 (treatment 1)	Hyaluronic acid (HA) gel
T2 (treatment 2)	Probiotic *Limosilactobacillus reuteri* gel
T3 (treatment 3)	HA gel followed by *L. reuteri* gel
T4 (treatment 4)	Povidone-iodine (PVP-I)
